# Collinear facilitation and contour integration in autism: evidence for atypical visual integration

**DOI:** 10.3389/fnhum.2015.00115

**Published:** 2015-03-10

**Authors:** Stephen Jachim, Paul A. Warren, Niall McLoughlin, Emma Gowen

**Affiliations:** ^1^Faculty of Life Sciences, University of ManchesterManchester, UK; ^2^Psychological Sciences, University of ManchesterManchester, UK

**Keywords:** autism, Asperger’s Syndrome, collinear facilitation, contour integration, enhanced perceptual functioning, weak central coherence

## Abstract

Autism spectrum disorder (ASD) is a neurodevelopmental disorder characterized by impaired social interaction, atypical communication and a restricted repertoire of interests and activities. Altered sensory and perceptual experiences are also common, and a notable perceptual difference between individuals with ASD and controls is their superior performance in visual tasks where it may be beneficial to ignore global context. This superiority may be the result of atypical integrative processing. To explore this claim we investigated visual integration in adults with ASD (diagnosed with Asperger’s Syndrome) using two psychophysical tasks thought to rely on integrative processing—collinear facilitation and contour integration. We measured collinear facilitation at different flanker orientation offsets and contour integration for both open and closed contours. Our results indicate that compared to matched controls, ASD participants show (i) reduced collinear facilitation, despite equivalent performance without flankers; and (ii) less benefit from closed contours in contour integration. These results indicate weaker visuospatial integration in adults with ASD and suggest that further studies using these types of paradigms would provide knowledge on how contextual processing is altered in ASD.

## Introduction

Autism spectrum disorder (ASD) is a neurodevelopmental disorder characterized by deficits in social communication and social interaction as well as repetitive and restricted behavior. Although diagnosed primarily by social symptoms (Lord et al., 2000), autism is often accompanied by unusual sensory experiences that can affect individual or multiple modalities. These can include hypersensitivity such as an aversion to bright or flashing lights or hyposensitivity, where the individual seems to be unaware or slow to respond to a stimulus that should normally elicit a response (Bogdashina, 2003). The importance of sensory symptoms is highlighted by their recent inclusion in the Diagnostic and Statistical Manual of Mental Disorders (American Psychiatric Association, 2013). Alongside these sensory symptoms, instances of perceptual superiority have also been reported, such as superior performance in visual search tasks where it may be beneficial to ignore global context (for reviews see Dakin and Frith, 2005; Simmons et al., 2009). Typically, in such tasks participants locate a particular stimulus or shape hidden among other “distractor” stimuli that form part of a larger, global scene while behavioral measures such as reaction times or error rates are taken to indicate performance. For example, ASD participants are often faster than neuro-typicals (NTs) at finding simple shapes embedded in more complex figures (Shah and Frith, 1983). This may be the result of perceiving an object in a dis-integrated way—seeing it in terms of its “parts” rather than as a “whole” leading to the possibility that the perceptual advantage observed in ASD participants result from altered visual integration (Happé, 1996). Visual integration is a broad concept, but the current work focusses on the ability to integrate local information from different parts of a visual image into a larger, global percept such as a contour or shape. At the neurophysiological level, there is a large body of evidence that visual integration is mediated by interactions that occur between visual neurons, involving horizontal, feedforward and feedback connections (Kapadia et al., 2000; Angelucci et al., 2002; Nurminen and Angelucci, 2014). Visual neurons in early visual cortex (V1) are not only influenced by direct feedforward input from the environment, but also by horizontal (lateral) connections from neighboring cells and feedback from higher cortical areas (Shushruth et al., 2013). As this “contextual modulation” contributes to the detection, extraction and separation of shapes and objects from the background (Gheorghiu et al., 2014; Nurminen and Angelucci, 2014; Schmid and Victor, 2014) it is particularly relevant for understanding why perception is atypical in autism. Here we explore visual integration in ASD using two well-established psychophysical tasks that rely on contextual modulation: collinear facilitation and contour integration (for an in depth review of these tasks in relation to shape processing see Loffler (2008)).

**Figure 1:**
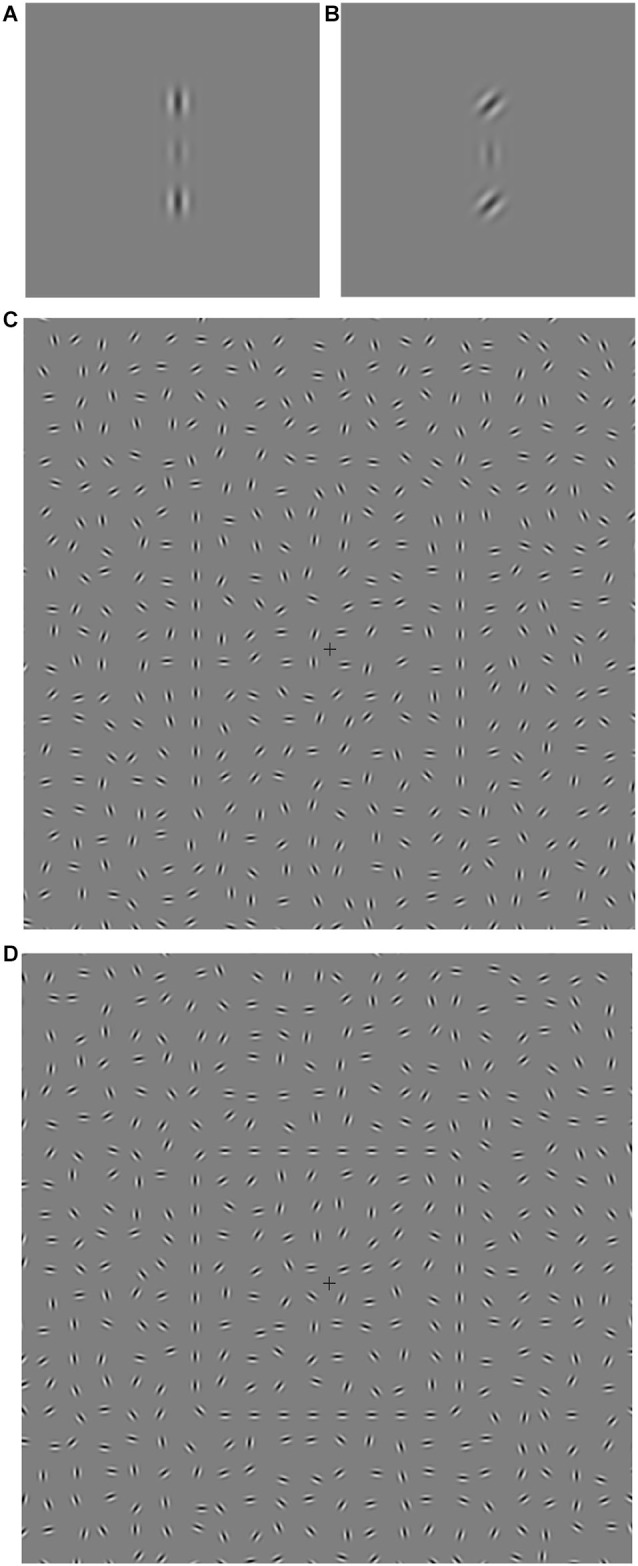
**(A–D)** Central area screenshots of the stimuli used for Experiment 1 (collinear facilitation, **A,B**) and Experiment 2 (contour integration, **C,D**). **(A)** Flankers are in the vertically aligned condition (0° orientation offset). The separation between the central target and each vertically aligned flanker is 3λ (one λ is equal to one Gabor cycle or wavelength, i.e., the combined width of a single light and dark stripe). **(B)** An example of one of the three orientation offset conditions, in this case the flanker offset is 45°. **(C)** Open contour: lines made up of aligned Gabor elements are embedded in a background of randomly oriented Gabors. **(D)** Closed contour: a square made up of aligned Gabor elements is embedded in a background of randomly oriented Gabors.

In their seminal demonstration of collinear facilitation, Polat and Sagi (1993) showed that a faint Gabor target (a sinusoidal grating modulated by a Gaussian window) was easier to detect when flanked by high contrast, co-aligned Gabors compared to a no flanker (baseline) condition (Figure 1A). This phenomenon is critically dependent on several parameters including the orientation offset of the flankers relative to the target Gabor and the distance between the flankers and the target. Collinear facilitation is strongest when the target and flankers are co-aligned (0° offset, Figure 1A) compared to when the orientation of the flankers and target are offset (Figure 1B). In addition, collinear facilitation is strongest when the target and flankers are separated by approximately 3λ (1λ = 1 wavelength, the combined width of a single light and dark Gabor stripe), decreasing at larger distances, with suppression occurring for distances <2λ. Collinear facilitation is thought to be mediated by propagation of excitatory signals from flanker to target cells along intrinsic horizontal (lateral) connections in V1 (Rockland and Lund, 1982; Gilbert and Wiesel, 1983; Livingstone and Hubel, 1984; Gilbert and Wiesel, 1989; Polat and Sagi, 1993). These connections are more numerous between cells of similar orientation preference, which could account for greater collinear facilitation found when the target and flankers are aligned (Kapadia et al., 1995; Fitzpatrick, 1996; Stettler et al., 2002). However, when target and flanker stimuli are positioned <2λ apart, suppression occurs via the horizontal connections, termed lateral inhibition (Hartline, 1949; Blakemore et al., 1970; Polat and Sagi, 1993). There is also likely to be feedback from higher cortical areas influencing contextual effects in V1 (Hupé et al., 1998; Angelucci et al., 2002; Freeman et al., 2003; Huang and Hess, 2008). Feedback projections may enhance collinear contour elements by excitation (Shmuel et al., 2005), or by attentional modulation (Freeman et al., 2004).

Only one previous study has investigated collinear facilitation in autism (Kéïta et al., 2011). They found more facilitation for an autistic group than a control group when collinear flankers were separated from the target Gabor by 3λ. The authors argued that these findings support altered lateral connectivity in autism, which in turn could lead to atypical responses to contextual information and be partly responsible for enhanced perceptual functioning in ASD. In order to extend these findings, our first aim was to examine collinear facilitation in adults with autism but using a different flanker condition: Instead of distance, we used four flanker orientation offsets. Flanker orientation was chosen as it is a well-tested and reliable paradigm in the neuroptypical literature and can provide a detailed picture of the pattern of lateral connectivity as the strength of collinear facilitation is likely to result from different levels of connectivity between cells of different orientation preference (Kapadia et al., 1995; Fitzpatrick, 1996; Stettler et al., 2002). In line with the findings of Kéïta et al. (2011), we predicted altered visual integration, demonstrated by higher collinear facilitation at the four flanker orientation offsets compared to the control group.

Our second aim was to examine visual integration using a Gabor based contour integration task. Contour integration involves the detection of Gabor elements that form a contour amidst a background of randomly oriented Gabors (Field et al., 1993; Figure 1C). Target detection is impaired when the relative orientation (jitter) of adjacent path elements is increased. Contours can either be open (e.g., a single line) or closed (e.g., a shape). Interestingly, Kovács and Julesz (1993) found that closed contours were easier to detect than non-closed contours, an effect they ascribe to the *global stimulus structure* of the closed contour. Later evidence further supports the idea of a separate global closure driven mechanism that is sensitive to the detection of closed contours (Mathes and Fahle, 2007; Gerhardstein et al., 2012). Like collinear facilitation, contour integration is thought to involve excitatory horizontal connections between cells of similar orientation preference within V1 (Rockland and Lund, 1982; Gilbert and Wiesel, 1983, 1989; Livingstone and Hubel, 1984; Field et al., 1993; Kovács and Julesz, 1993; Pettet et al., 1998; Tversky et al., 2004), and/or feedback from extrastriate areas (Hupé et al., 1998; Angelucci et al., 2002; Achtman et al., 2003; Altmann et al., 2003; Shpaner et al., 2013). Such feedback is likely to originate from areas responsible for simple shape processing such as the lateral occipital complex (LOC; Mijović et al., 2014) and may contribute to enhanced detection of closed contours (Gerhardstein et al., 2012). For example, when human observers viewed line drawings that varied from open lines to 2D and 3D shapes, the progression from open to closed stimuli was positively correlated with fMRI brain activity in the LOC, and negatively correlated with activity in V1 (Murray et al., 2002).

Contour integration provides a useful test of visual integration as it involves combining a number of separate elements into a larger, global percept, contributing to the grouping and segmentation of objects in the environment (Loffler, 2008). A number of studies have previously investigated contour integration in individuals with ASD, and they report conflicting results. Blake et al. (2003) asked children to identify the quadrant in which a circular shape composed of “line” stimuli was located and found no difference in contour detection between the autistic and control group. However, these line stimuli have been criticized for containing low spatial frequencies enabling grouping to occur via a low spatial frequency mechanism, as opposed to true grouping mechanisms (Dakin and Frith, 2005). More recent studies overcome this confound by using band-pass Gabor stimuli. Del Viva et al. (2006) used a computerized display of random Gabor distractors to test how well participants could locate the quadrant in which a circular Gabor contour was placed. They found equivalent performance when comparing autistic children to age-matched controls. Kemner et al. (2007) used a card-based version of the contour integration task with closed contour stimuli, again finding similar performance between autistic children and control groups. One limitation of these studies is that they use long stimulus presentation times (>1 s), which may have hidden any differences if the ASD group required more time to distinguish the shape (Van der Hallen et al., 2014). More recent work has identified group differences. In an electrophysiological study, Pei et al. (2009) recorded visually evoked potentials while children watched a Gabor display that alternated every 500 ms between circular contours and random patterns. They failed to detect what they considered to be the neural correlate of contour integration in low functioning children with autism, suggesting that reduced contour integration may be present for shorter stimulus durations. Evers et al. (2014) reported that children with ASD were slower and less accurate than controls at identifying contours based on everyday objects, which gradually emerged from a background of randomly oriented Gabors.

These latter studies suggest that atypical contour integration in ASD may exist, but is only apparent when using more challenging protocols such as shorter stimulus durations, or object identification as opposed to detection of simple shapes. At present, it remains unclear whether alterations in contour integration can be found for more simple shape detection when using shorter presentation times. Furthermore, no study to date has compared open vs. closed shapes in individuals with ASD. This is potentially important given that the detection of closed contours may involve a separate global closure driven mechanism compared to open contours, and that integrating parts into a whole may be problematic for ASD (Happé and Frith, 2006; Van der Hallen et al., 2014). Finally, all previous studies examining contour integration in ASD have been performed on children, but we chose to study adults in order to use a more standardized, controlled psychophysical set up, as has been used previously with neurotypical adults (Field et al., 1993; Dakin and Baruch, 2009; Schumacher et al., 2011). Therefore, in the current study we assessed contour integration using relatively simple open and closed stimuli but with short presentation times. These two stimuli were presented with their Gabors arranged at different levels of orientation (jitter) ranging from 0 degrees (contour is easily detectable) to ±90 degrees (contour is difficult to detect). Contour integration ability was measured by determining the level of jitter at which a participant could successfully detect the target with 75% accuracy, termed jitter tolerance. If participants with ASD have impairments in visual integration, then we would expect weaker contour integration when compared to controls, particularly for closed contours.

## Materials and Methods

### Participants

Thirteen ASD participants (3 female) and thirteen healthy controls matched on age, sex, handedness and full scale IQ were recruited through local support groups and volunteer advertisements. This sample number was based on a pilot study examining collinear facilitation and contour integration in 17 neurotypical participants using G*power 6 (Faul et al., 2007). For the collinear facilitation experiment we calculated power for the main effect of orientation. There was an effect size of 1.13 and power of 0.99, giving a sample size of 13. For the contour integration experiment we calculated power for the difference between lines and squares. There was an effect size of 1.65 and power of 0.99, giving a sample size of 9. We were unable to do a power calculation for the autistic group, as this is the first time these particular paradigms have been used. However, a *post hoc* power test using our autistic participants indicates a power of 1 for the above comparisons.

ASD participants had been given a diagnosis by outside clinical assessment (World Health Organization, 1993; American Psychiatric Association, 1994) of Asperger’s syndrome. Diagnosis was confirmed using module 4 of the Autism Diagnostic Observation Schedule by a qualified researcher (Lord et al., 2000). Demographics of the individual participants can be seen in Table 1. One participant fell just below the cutoff for ASD, likely due to his older age facilitating the development of compensatory strategies, which can make ADOS scoring more challenging. However, their data was included as statistical comparisons remained significant and in the same direction when this participant was removed. Age, Full Scale, Verbal and Performance IQ, measured using Wechsler Abbreviated Scale of Intelligence (Wechsler, 1999) did not differ significantly between the two groups (Table 1). Four of the participants in each group were left-handed, all had normal or corrected-to-normal vision (6/6), using reduced Snellens at 33 cms, with a lower limit of 6/6. All had 60′ stereo acuity measured using the TNO stereotest. Participants gave written informed consent and the study was approved by the University of Manchester Research Ethics Committee.

**Table 1:** **Participant demographics**.

ASD	Age	Sex	Hand	FSIQ	PIQ	VIQ	ADOS	Controls	Age	Sex	Hand	FSIQ	PIQ	VIQ
1	25	F	R	128	108	142	7	1	38	M	R	129	128	132
2	18	M	L	117	119	110	7	2	20	M	R	127	111	136
3	40	M	R	134	121	138	10	3	34	F	R	136	127	136
4	30	F	R	131	119	136	8	4	19	M	L	133	123	136
5	42	M	L	123	120	119	5	5	29	F	R	124	121	120
6	38	M	L	134	132	128	10	6	36	M	R	124	132	110
7	39	M	R	118	116	117	7	7	22	M	R	114	128	102
8	21	M	R	109	100	116	8	8	40	M	R	107	97	118
9	39	M	L	101	99	102	10	9	20	F	L	104	106	101
10	23	M	R	132	128	130	10	10	20	M	R	121	123	115
11	22	M	R	118	111	119	9	11	26	M	R	138	126	141
12	22	M	R	118	119	114	10	12	38	M	L	118	119	103
13	20	F	R	105	102	106	10	13	34	M	L	123	119	120
Mean	29.15			120.62	114.92	121.31		Mean	28.92			122.92	120.00	120.77
SD	9.07			11.00	10.34	12.55		SD	8.08			10.31	9.93	14.28
Group	*t* = 0.07;			*t* = −0.55;	*t* = −1.28;	*t* = 0.102;		
comparison	*p* = 0.95			*p* = 0.625	*p* = 0.665	*p* = 0.581

*Statistical comparison between the two groups on age, Full Scale IQ (FSIQ), Performance IQ (PIQ) and Verbal IQ (VIQ) can be seen in bottom row*.

### Apparatus

Stimuli for all experiments were displayed on an Iiyama MA203DT CRT monitor with a screen resolution of 1600 × 1200 and a vertical refresh rate of 85 Hz. Participants were seated in a darkened room with the viewing distance to screen fixed at 70 cm by the use of a chinrest. The mean display luminance for all experiments was 42 cd/m^2^. A video-switcher device gave true 12-bit luminance resolution (Li et al., 2003). The video output was gamma corrected using a psychophysical procedure described by Li et al. (2003) and calibrated with a Minolta CS-100 Chroma meter colorimeter (Konica Minolta Sensing America Inc., Ramsey, New Jersey). Participant responses were collected via two color-coded keys on a standard UK computer keyboard.

### Stimuli

For Experiment 1, stimuli consisted of target and flanker Gabors generated with Psychtoolbox (Brainard, 1997; Pelli, 1997; Figure 1). Each Gabor had a bandwidth of 1.14 octaves, standard deviation of 0.16° and a spatial frequency of 3.0 cpd. The target Gabor was presented centrally and flanked vertically by two high contrast Gabors (60% Michelson contrast) with a separation of 3λ (1° of visual angle). The orientation offset of the flanking Gabor pair (0°, 15°, 30° or 45°) gave four experimental conditions plus a baseline (no flankers) condition.

For Experiment 2, stimuli consisting of contour and background Gabors were generated and displayed by a Java program. Each Gabor had a bandwidth of 1.2 octaves, standard deviation of 0.1° and a spatial frequency of 4.6 cpd. All Gabors were displayed at 100% Michelson contrast. The display was divided into a 33 × 24 invisible grid and each cell initially contained a randomly placed Gabor. The Gabor position was perturbed ±*x* pixels horizontally and ±*y* pixels vertically from the center of the cell, where *x* and *y* represent a pair of random integers independently sampled from a uniform distribution over the range [0, 11]. Each Gabor was randomly oriented (jittered) within the range ±90°. Experiment 2 used two different target stimuli; vertical lines (Figure 1C) and a square (Figure 1D). For the lines stimulus, the vertical columns of 10 Gabors 5° to the left and right of the central fixation cross were replaced by columns of 10 Gabors that were centrally located within the cells to form a straight line. The jitter of the target Gabors varied from trial to trial. A comparison stimulus was created in the same way, but each of the Gabors in the two 10 Gabor columns were jittered within the range ±90° but still centrally located within the cells. This ensured that the only difference between the target presentation and the comparison presentation was the jitter of the Gabors that formed the target contour lines. The square stimulus (and square comparison) were formed in the same way, with the addition of two horizontal lines, and ±45° angled corner Gabors. The spatial location of the vertical lines was identical for both the lines and square stimuli and remained constant throughout the experiment. During each trial, all the Gabors forming the target contour were jittered by an amount from the uniformly distributed ranges 0°, 0–15°, 0–30°, 0–45°, 0–60°, 0–75° or 0–90° giving seven jitter conditions.

### Procedure

**Figure 2:**
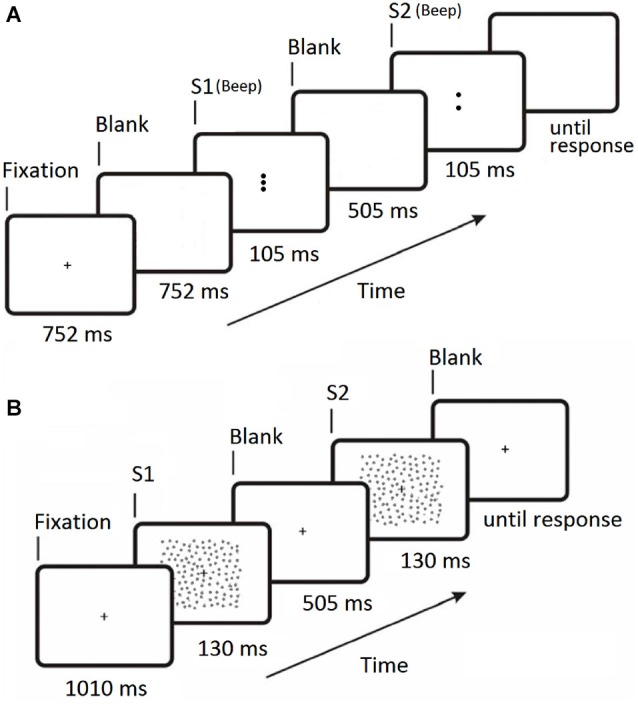
**Timelines for the two 2IFC trial sequences. (A)** Experiment 1 (collinear facilitation). S1 is the first stimulus presentation period, S2 is the second stimulus presentation period. The central Gabor target appears randomly in either S1 or S2 on each trial and a 100 ms beep was sounded at the start of both S1 and S2. **(B)** Experiment 2 (contour integration). S1 is the first stimulus presentation period, S2 is the second stimulus presentation period. The contour target appears randomly in either S1 or S2 on each trial. For Experiment 2 the fixation cross remains on screen at all times.

All experiments used a two interval forced choice (2IFC) procedure and stimuli were viewed binocularly. The trial sequence for Experiment 1 is shown in Figure 2A. Each stimulus presentation had a duration of 105 ms and was accompanied by an audible beep. Either the first or second stimulus presentation contained the central target Gabor and the participant indicated which interval the target appeared in by pressing one of two keys on the keyboard. Each of the five conditions (four flanker orientation offsets and a no-flanker baseline condition) were randomly interleaved during the experiment, each condition occurring once every five trials. Each condition was presented 60 times, giving a total of 300 trials. Participant target contrast thresholds were determined by varying the target contrast using the psi-method (Kontsevich and Tyler, 1999) as implemented by the Palamedes Matlab toolbox (Kingdom and Prins, 2009).

The trial sequence for Experiment 2 is shown in Figure 2B. Participants were instructed to focus on the fixation cross (which was always visible) throughout the entire experiment. The two stimulus presentations were presented for 130 ms each, separated by a blank screen containing the fixation cross for 505 ms. Either the first or second stimulus presentation contained the target (Figure 1C), and the participant indicated which interval the target appeared in by pressing one of two keys on the keyboard. There were forty randomly ordered presentations of each of the seven jitter ranges, presented in four blocks of 70 trials. The participant’s ability to tolerate jitter was determined using the method of constant stimuli (Field et al., 1993; Schumacher et al., 2011). After a short break the procedure was repeated using the square stimulus (Figure 1D). The order of the two stimuli was counter-balanced across participants.

As the contour integration experiment uses peripheral stimuli that might encourage saccades away from the central fixation cross, the left eye of each participant was tracked using GazeTracker software (San Agustin et al., 2010) and a remotely located camera. Nine point calibration with GazeTracker gave a spatial resolution of 0.25°, and eye movements were recorded and displayed on the experimenter’s monitor using a custom Java application. Participants were informed by the experimenter if their gaze-point deviated from the fixation cross by more than 1.5° in any direction. Eye-tracking co-ordinates for 84.3% of the trials were successfully recorded. For the ASD group, fixation for 86.4% of trials was within ±1.5° of cross. For the NT group, fixation for 94.3% of trials was within ±1.5° of the cross. Analysis performed after dropping trials recorded outside the fixation area did not significantly affect the results. We therefore used data from all trials in the reported analysis.

### Analysis

For Experiment 1, the psi-method returned the running estimates of the contrast thresholds (Weibull distribution; 81.6% correct performance level) based on the posterior distribution for each of the five flanker conditions. Facilitation is measured in decibel units (dB), which are −20 × log_10_ value of the contrast ratio, the contrast ratios being defined as the threshold at each flanker orientation offset divided by the baseline threshold (Polat and Sagi, 1994). For Experiment 2 which used the method of constant stimuli, we fitted a logistic psychometric function using the Psignifit Matlab toolbox (version 2.5.6),^1^ which is based on Wichmann and Hill (2001). Performance over the seven jitter levels was determined using maximum-likelihood estimation. Thresholds were estimated at the 75% correct performance level. Correlations were performed between the two contour integration tasks and between the contour integration and collinear facilitation tasks.

### Experiment 3

**Figure 3:**
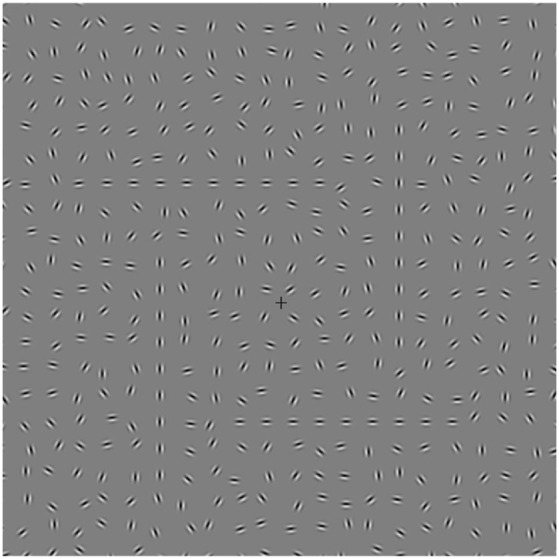
**Central area screenshot of experiment 3 showing the “windmill” contour embedded in a background of randomly oriented Gabors**. This stimulus had the same number of Gabors as the square contour stimulus but with an “open” contour arrangement.

In a third experiment, we tested whether the improvement predicted with the square compared to lines stimulus was caused by a closed contour (Kovács and Julesz, 1993), rather than simply an increase in the number of GPs in the target (and therefore a more powerful stimulus). We repeated Experiment 2 with an additional stimulus (Figure 3). This “windmill” stimulus tested for an open contour condition having the same number of GPs as the square. We predicted that the windmill will be easier to detect than the lines, but harder to detect than the square. Stimuli were tested on a new NT group (*n* = 15 (8 female), mean age (±SD) 20.7 ± 2.3). All had normal or corrected-to-normal vision using reduced Snellens, and 60 s arc stereo acuity measured using the TNO stereotest.

## Results

### Collinear Facilitation

**Figure 4:**
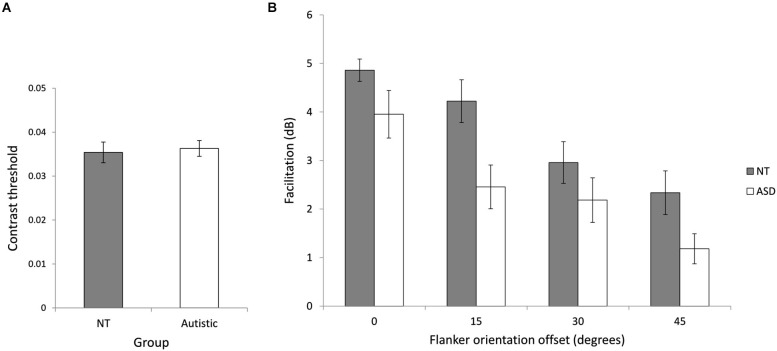
**Experiment 1 (collinear facilitation). (A)** Baseline contrast thresholds for the two groups. **(B)** The relationship between flanker orientation offset in degrees and mean facilitation in decibels for NT and ASD subjects. Error bars indicate *SE*. Facilitation is reduced for both groups with increasing flanker orientation offset and NT performance was significantly higher across flanker condition.

We investigated collinear facilitation for the ASD and NT groups by measuring facilitation at four different flanker orientation offsets. The NT (*M* = 0.035, *SD* = 0.008) and ASD (*M* = 0.036, *SD* = 0.007) baseline contrast thresholds were not significantly different (*t*_(24)_ = 0.28, *p* = 0.78), indicating similar contrast sensitivity between the two groups (Figure 4A). Figure 4B shows the mean facilitation across the groups at different flanker orientation offsets. Consistent with previous studies, facilitation is highest when the flankers are vertically aligned with the target (0° orientation offset) and decreases with increasing flanker orientation offset. A mixed ANOVA of group (NT and ASD) by flanker orientation offset (0°, 15°, 30° and 45°) revealed a significant main effect of flanker orientation offset (*F*_(3,72)_ = 33.11, *p* < 0.001, $\eta_{P}^{2}$ = 0.56) showing that overall, facilitation decreased as orientation offset increased [0° (*M* = 4.41, *SD* = 1.44), 15° (*M* = 3.34, *SD* = 1.80), 30° (*M* = 2.57, *SD* = 1.62), 45° (*M* = 1.76, *SD* = 1.48)]. There was also a significant main effect of group (*F*_(1,24)_ = 5.65, *p* = 0.021, $\eta_{P}^{2}$ = 0.20) indicating that the NT group showed more facilitation than the ASD group [NT (*M* = 3.56, *SD* = 1.72), ASD (*M* = 2.41, *SD* = 1.81)]. *Post-hoc* paired *t*-tests (Bonferroni corrected) were performed, examining facilitation at each orientation between the two groups. They revealed a significant difference at an offset of 15°, [NT (*M* = 4.32, *SD* = 1.60), ASD (*M* = 2.46, *SD* = 1.61), (*t*_(23)_ = 2.89, *p* = 0.032)], indicating higher facilitation for NTs at this offset.The group × orientation offset interaction was not significant (*F*_(3,72)_ = 1.80, *p* = 0.34, $\eta_{P}^{2}$ = 0.05. This result contradicts previous findings of higher facilitation in ASD (Kéïta et al., 2011), but supports the idea of atypical integration in autism.

### Contour Integration

**Figure 5:**
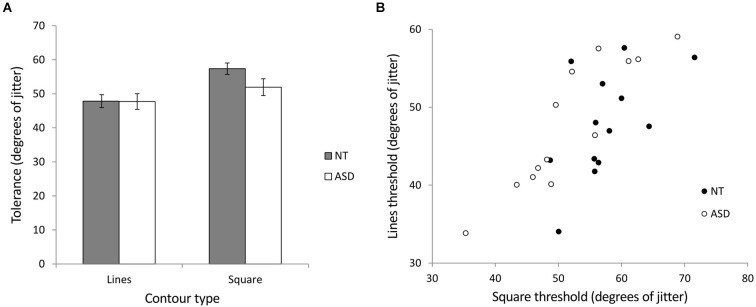
**Experiment 2 (contour integration). (A)** The relationship between jitter tolerance (the level of jitter at which a participant could successfully detect the target with 75% accuracy) and the detection of open (lines) and closed (square) figures for NT and ASD subjects. Higher values indicate better detection performance. The NT group show a larger difference in jitter tolerance between lines and squares as indicated by a significant group × contour-type interaction. Error bars indicate *SE*. **(B)** Scatterplot showing the correlation between contour integration performance (tolerance to jitter, higher is better) for lines and square stimuli for the NT and ASD groups. The correlation was stronger for the ASD group (*r* = 0.90) compared to the NT group (*r* = 0.56) which may suggest similar mechanisms underlying the processing of open (lines) and closed (square) stimuli in the case of the ASD group.

We investigated contour integration by calculating jitter tolerance in degrees (the level of jitter at which a participant could successfully detect the target with 75% accuracy) for the ASD and NT groups in two stimulus conditions (lines and squares). Figure 5A shows the mean jitter tolerance across the groups for the different stimuli. A mixed ANOVA of group (NT and ASD) by stimulus (lines and square) revealed a significant main effect of stimulus (*F*_(1,24)_ = 46.26, *p* < 0.001, $\eta_{P}^{2}$ = 0.66) indicating that, In line with previous studies, detection was better for closed compared to open stimuli. There was no main effect of group (*F*_(1,24)_ = 0.98, *p* = 0.33, $\eta_{P}^{2}$ = 0.04) but crucially, we found a significant interaction of group × stimulus (*F*_(1,24)_ = 7.00, *p* = 0.014, $\eta_{P}^{2}$ = 0.23). Further investigation of the interaction showed that NT participants could tolerate significantly more jitter with the square stimulus (*M* = 57.38°, *SD* = 6.01) than the line stimulus (*M* = 47.83°, *SD* = 6.86; *t*_(12)_ = 5.64, *p* < 0.001). Although ASD participants could also tolerate significantly more jitter with the square stimulus (*M* = 51.93°, *SD* = 8.95) than the line stimulus (*M* = 47.73°, *SD* = 8.28; *t*_(12)_ = 3.80, *p* = 0.003), this improvement was significantly less than that shown by the NT group, a finding which may indicate reduced visual integration in autism. Simple main effects comparison indicated that the ASD and NT group did not significantly differ for the lines (*t*_(24)_ = 0.036, *p* = 0.972) or square (*t*_(24)_ = 1.82, *p* = 0.081) stimuli. Pearson correlation coefficients were used to examine any relationship between the processing of the two stimuli. Both the NT (*r* = 0.56, *p* = 0.048) and the ASD (*r* = 0.90, *p* < 0.001) groups showed significant correlations between the integration of the lines and the integration of the square (Figure 5B). In order to directly compare these correlation coefficients between the two groups, the correlation coefficients were converted to z-scores using Fisher’s r-to-z transformation (Fisher, 1915). Comparison of these z scores (Cohen and Cohen, 1983) revealed that the group difference between these r-values just missed significance (*Z* = 1.88; *p* = 0.06).

**Figure 6:**
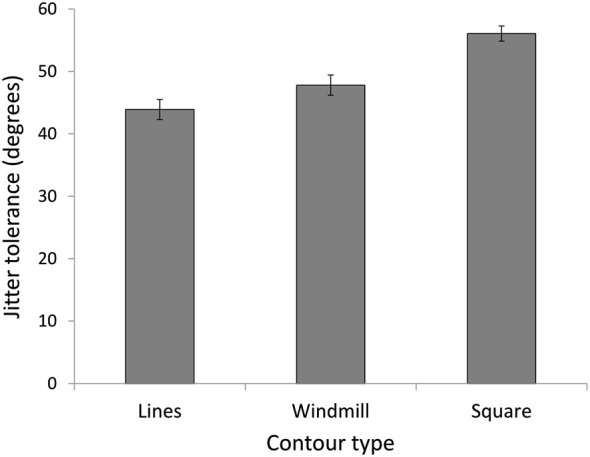
**Experiment 3.** Jitter tolerance in degrees at which NT participants could detect the stimuli with 75% accuracy. Results confirm predictions of easier detection for a closed contour (square), even when the open contour (windmill) had the same number of elements as the closed contour (square).

In experiment 3, we investigated CI for three stimulus conditions (lines, windmill, square) in a new group of NT participants, to assess whether the increased detection for the NT group with the square stimulus in Expt. 2 was was the result of global enhancement or simply more stimulus information. A repeated measures ANOVA showed a main effect for stimulus (*F*_(3,42)_ = 49.05, *p* = 0.000, $\eta_{P}^{2}$ = 0.78) (Figure 6). As predicted, the windmill (*M* = 47.80, *SD* = 6.28), was easier to identify than the lines (*M* = 43.90, *SD* = 6.18; *t*_(14)_ = 3.32, *p* = 0.015) but harder to identify than the square (*M* = 56.07, *SD* = 4.69; *t*_(14)_ = 5.83, *p* < 0.0001).

## Discussion

We investigated visuospatial integration in participants with ASD by examining collinear facilitation at four flanker orientation offsets, and contour integration for open and closed contours. In support of altered integration, results indicated that collinear facilitation was reduced for the ASD group, and that the detection advantage for closed contours was smaller for the ASD group.

For the collinear facilitation experiment, both our groups demonstrated the usual pattern of performance, showing decreasing facilitation with increasing flanker orientation offset. However the ASD group demonstrated reduced collinear facilitation compared to the control group, which was significant for the 15° offset. As contrast sensitivities for the target in the baseline (no flanker) condition were similar for both groups, reduced collinear facilitation in the ASD group cannot be attributed to decreased visual acuity. The baseline condition is consistent with previous findings suggesting no difference in visual acuity (Falkmer et al., 2011; Albrecht et al., 2014) or contrast sensitivity (Koh et al., 2010) for equivalent spatial frequencies between ASD and NT groups.

Our results showing reduced or equivalent collinear facilitation contradict those of Kéïta et al. (2011) who reported increased collinear facilitation at 3λ for adults with ASD relative to controls. As ASD is a heterogeneous condition it is possible that the two studies are testing different subgroups within the population. Indeed, our participant group is composed of individuals diagnosed with Asperger’s and has a much higher IQ compared to those in the study by Kéïta et al. (120 vs. 100) which excluded Asperger’s. However, there are also several differences between our study and that of Kéïta et al. (2011). For example, Kéïta et al. used a longer stimulus presentation time (500 ms compared to 100 ms) and added grayscale noise to the target and flankers, a manipulation that has been shown to reduce facilitation (Huang and Hess, 2007). The addition of grayscale noise may account for the similar levels of facilitation found by Kéïta et al. at both 6λ and 3λ flanker conditions for controls, a result that contradicts a substantial body of previous collinear facilitation studies indicating greater facilitation at 3λ (Polat and Sagi, 1993, 1994, 2006, 2007; Polat and Norcia, 1996; Adini et al., 1997; Polat, 1999; Huang et al., 2006; Huang and Hess, 2007; Sterkin et al., 2009). In contrast, our NT results are consistent with levels observed at 3λ separations in these previous studies. The current findings raise the possibility that the ASD group in Kéïta et al. (2011) were less affected by the noise than controls, but future work comparing flanker separations with and without noise is required.

If collinear facilitation is assumed to rely on horizontal connectivity in V1 for the integration of the flanker signals at the target site (Polat and Sagi, 2007), then reduced facilitation could be the result of fewer, longer or slower connections between flanking and target neurons. Morphological studies however, suggest a bias towards shorter V1 connections in autism (Casanova et al., 2006). One possible explanation for the reduced facilitation shown by participants with ASD could lie with the temporal integration of the visual stimuli. Although the target and flankers appear onscreen simultaneously, there are two sources of delay that impact integration of the flanker and target signals in V1. Firstly, because of temporal coding of contrast (Reich et al., 2001) and the low contrast of the target stimuli, target neurons respond later than flanker neurons to the visual input. Secondly, signals from the flanker sites take time to reach the target site because of the relatively slow propagation rate of signals along horizontal connections (Bringuier et al., 1999; Cass and Spehar, 2005). These delays determine the temporal window, or overlap, during which integration of signals from the flankers and target can occur. Differences in these delays between the ASD and NT groups may therefore contribute to the decrease in facilitation shown by the ASD group. This idea complements theories suggesting altered temporal processing in autism (Gepner and Féron, 2009) and evidence for poorer performance on temporal perception tasks (de Boer-Schellekens et al., 2013).

Altered top-down processing in autism (Frith, 2003) may also be a factor in reduced collinear facilitation. Huang and Hess (2008) showed that collinear facilitation occurred when the target was presented *before* the flankers, a result that is difficult to reconcile with the horizontal connectivity account given above (Polat and Sagi, 2006). They propose an additional facilitating mechanism based on rapid orientation-specific feedback from V2 (Girard et al., 2001). If this feedback is attenuated in ASD, then facilitation may be impaired. Another top-down process that can modulate collinear facilitation is attention; facilitation depends on whether or not the flankers are attended (Freeman et al., 2003). Therefore, reduced attentional feedback may also influence lateral interactions in V1 (Gilbert et al., 2000).

Turning to the contour integration experiment, both groups showed a significant improvement viewing the square stimulus, a finding consistent with previous work demonstrating easier detection of closed compared to open contours (Kovács and Julesz, 1993; Mathes and Fahle, 2007). However, the significant interaction revealed a greater effect of closure for the NT group, indicating that altered contour integration in ASD adults is apparent for the detection of simple shapes. Experiment 3 was designed to rule out the possibility that increased visual information (more Gabors) in the square compared to the lines may have driven the enhancement of the square and showed that while the windmill stimulus led to better contour detection than the lines, contour integration for the closed square stimulus was still better than the windmill. In line with previous results that have equated Gabor number between closed and open stimuli (Mathes and Fahle, 2007), these findings suggest that the number of Gabors do play a role, but that NT participants are also sensitive to the closed nature of the square stimulus. It remains possible that the presence of corners in the square compared to windmill may contribute to enhanced perception of the square due to “good continuation” rather than closure. Against this argument is evidence for a closure mechanism when factors such as good continuation have been controlled for (Gerhardstein et al., 2012).

There is some debate as to the exact mechanism behind the improvement in contour detection seen with closure. Low-level explanations suggest that this improvement results from the properties of the horizontal connections within V1 (Pettet et al., 1998; Yen and Finkel, 1998; Tversky et al., 2004). For example, facilitation between similarly orientated elements may be able to propagate multiple times around V1 cells representing the closed contour, enhancing contour integration, termed “reverberation” (Pettet et al., 1998; Yen and Finkel, 1998). However, in support of a distinct closure detection mechanism, Gerhardstein et al. (2012) showed that these low-level accounts cannot fully account for closure, and suggested the need for a separate global or semi-global mechanism, speculating on the role of V4 as a global shape processor (Pasupathy and Connor, 2002). Physiological evidence also points towards a top-down influence in the perception of closed contours. Using fMRI, Murray et al. (2002) studied brain activity whilst human subjects viewed simple line drawings of random lines, 2D shapes and 3D shapes. This progression from open to closed stimuli was positively correlated with activity in the LOC, and negatively correlated with activity in V1. The authors suggest that grouping-processes at higher levels generate feedback which reduces activity in lower areas. Furthermore, Gilad et al. (2013) trained macaques to perform a closed-contour integration task and monitored their V1 activity using voltage-sensitive dye imaging. They observed the initial brightening of V1 regions representing the contour Gabors, followed by dimming of V1 regions representing the background Gabors. Both of these events had the effect of increasing contour saliency. Gilad et al. suggest that the initial brightening is mediated by horizontal as well as feedback connections, whereas the later background suppression may be the result of changes in top-down feedback to V1.

Therefore, it is possible that the reduced effect of closure in the ASD group results from altered top down feedback, a suggestion that receives indirect support from two aspects of our findings. Firstly, the “lines” compared to “square” improvement shown by the ASD group is similar to the “lines” compared to “windmill” improvement demonstrated by the NT follow-up group. This comparison could be interpreted as the ASD group benefiting from the extra contour information in the square, but not receiving a top-down “boost” to further augment contour integration to NT levels. Secondly, the stronger correlation between the lines and the square results for the ASD group compared to NT group (Figure 5) was reaching significance, suggesting that similar processing mechanisms may be at work for both stimuli in the ASD group, but that different mechanisms (i.e., feedback) may also be operating in the NT group, when processing closed stimuli. A reduction in modulatory feedback is consistent with a predictive coding model of visual perception (Rao and Ballard, 1999), in which higher brain areas make predictions about input from lower brain areas, and then feedback these predictions to lower areas, where the predicted sensory information is compared to the actual sensory input. Altered predictive coding, such as the generation of less precise predictions has recently been forwarded as an explanation for the perceptual differences found in autism including the decrease in global processing (van Boxtel and Lu, 2013).

While our comparison between lines and squares has provided novel evidence for altered contour integration in ASD,^2^ no significant differences were found when directly comparing the participant groups for either the lines or the square stimuli, supporting previous psychophysical research investigating contour integration for simple shapes in autism (Del Viva et al., 2006; Kemner et al., 2007). One limitation of our work is that it lacked power to obtain significant group differences: In order to achieve a power of 0.8 for comparison of the square stimulus, 24 participants in each group would be required. Consequently, our results highlight that a lack of power may have also contributed to previous negative findings using between groups analysis, but that the interaction between open and closed contours reveals a much stronger effect. Evidence of group differences with larger group sizes has recently been reported (Evers et al., 2014), although as this used a recognition task it also raises the possibility that group differences in contour integration may become apparent with more complex tasks.

In summary, we employed two psychophysical paradigms, collinear facilitation and contour integration to investigate visual integration in adults with ASD. The ASD group showed reduced collinear facilitation and less improvement in contour detection between open and closed contours compared to the NT group. These results, together with previous studies using different psychophysical approaches (Vandenbroucke et al., 2009) indicate weaker visuospatial integration in adults with ASD, which may underlie perceptual superiority in tasks that benefit from ignoring contextual information.

## Author Contributions

All authors contributed towards the design, interpretation, writing and revision of the manuscript. SJ performed data collection, programming of experiments and analysis.

## Conflict of interest statement

The authors declare that the research was conducted in the absence of any commercial or financial relationships that could be construed as a potential conflict of interest.

## Footnotes

^1^see http://bootstrap-software.org/psignifit/

^2^An interaction refers to the comparison of within group differences NOT between group simple main effects (Nieuwenhuis et al., 2011). Our interaction is sufficient evidence to show that the groups differ and simple main effects analysis between groups is not necessary for interpretation of the interaction. We added the simple main effects comparison only to compare with previous behavioural contour integration experiments. Therefore, low power for simple main effects analysis does not detract from our principle finding of a significant interaction (with large effect size) between group and stimulus.
